# Evaluating large language models for evidence-based clinical question answering

**DOI:** 10.1016/j.patter.2026.101519

**Published:** 2026-03-30

**Authors:** Can Wang, Yiqun Chen

**Affiliations:** 1 Department of Biostatistics, Johns Hopkins University, Baltimore, MD 21205, USA; 2 Departments of Biostatistics and Computer Science, Johns Hopkins University, Baltimore, MD 21205, USA

**Keywords:** large language models, clinical question answering, biomedical NLP, evidence-based medicine, benchmark datasets, AI for medicine, AI evaluation

## Abstract

Large language models show potential in clinical applications, yet reliability for evidence-based medicine requires rigorous evaluation. We curated a multi-source benchmark with more than 20,000 question answering pairs from systematic reviews and clinical guidelines to assess performance on GPT-5, GPT-4o-mini, Claude 4, and DeepSeek-v3. Accuracy was highest with structured guidelines (90%), lower with narrative sources (70%), and lowest with systematic reviews (50%–60%). All models struggled with ambiguous evidence. We found that higher citation counts for source material correlated with increased accuracy and observed moderate geographic variation in performance. However, accuracy did not vary significantly by publication year or domain prevalence. Retrieval-augmented generation bolstered performance; providing the top three PubMed-retrieved articles yielded a 23% accuracy gain. These patterns were consistent across models, demonstrating that source clarity and targeted retrieval drive performance. We conclude that stratified evaluation and retrieval strategies are essential for ensuring factual alignment and reliability in high-stakes clinical decision-making.

## Highlights

• LLMs are more accurate on clinical guidelines than on systematic reviews
• All tested models show overconfidence when evidence is weak or absent
• Retrieval-augmented generation boosts clinical question-answering accuracy
• Findings hold across leading proprietary and open-weight LLM families

## The bigger picture

There is increasing interest in using artificial intelligence (AI) systems to support clinical decision-making, but their readiness has mostly been measured using well-established facts gleaned from medical exams and textbook questions. In practice, medicine often rests on evidence that is incomplete, conflicting, or context dependent. There is therefore a need for tools that probe whether AI can handle real-world medical knowledge beyond the books. Systematic reviews and clinical guidelines offer a rich alternative. They capture the full spectrum of evidence quality, from strong, unambiguous recommendations to weak or absent findings; span wide medical domains; and carry a higher signal than individual publications, since human experts curate and review the underlying evidence.

We tested leading AI models on 20,000 questions synthesized from over 8,000 systematic reviews and clinical guidelines. Models still struggle with ambiguous evidence, but retrieval-augmented approaches substantially improve accuracy. As AI systems continue to be trained on larger and newer data, scalable evaluation pipelines grounded in real-world knowledge will become critical for determining whether AI is truly ready for high-stakes deployment in medicine.

## Graphical abstract

**Figure:**
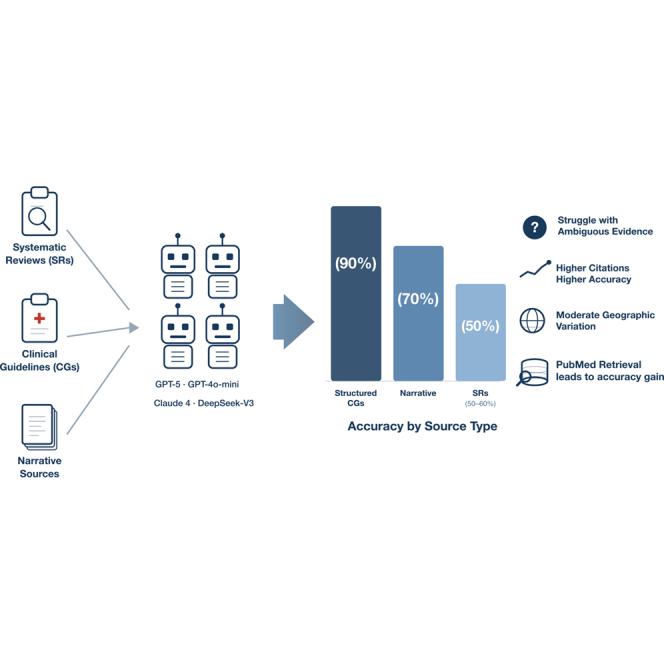

## Teaser

Can AI systems reliably navigate the complexity and uncertainty of real-world medical evidence? Wang and Chen evaluate leading AI models on 20,000 questions synthesized from over 8,000 systematic reviews and clinical guidelines, finding that models struggle when underlying studies show high effect-size variance or limited citation support.

## Introduction

Large language models (LLMs) have demonstrated strong capabilities in open-domain and medical question answering (QA) and reasoning,^1^^,^^2^ but their performance in complex, evidence-based clinical domains remains an active area of exploration.^2^ While prior benchmarks have evaluated biomedical QA performance across various formats,^3^^,^^4^^,^^5^ most existing datasets are derived from well-established medical practice and standardized questions (e.g., MedQA from medical licensing exams). The *transportability* of these QA datasets to real-world clinical practice has recently been called into question.^6^^,^^7^ This has spurred growing interest in whether LLMs can accurately address clinical questions grounded in diverse sources of evidence, particularly in settings that require reasoning about evidence quality.

In particular, many clinically relevant questions are difficult to characterize because the underlying evidence may be missing or contradictory (e.g., differing results from clinical trials vs. observational studies). Moreover, such information is not readily available in standalone test-style datasets (e.g., medical board exams), as the body of clinical evidence evolves continuously, whereas exam questions (1) are broader in scope and omit fine-grained diagnostic reasoning and (2) typically lag by several years. A key avenue for capturing such evidence is through systematic reviews, which are widely regarded as the gold standard for evidence-based medicine. Systematic reviews comprehensively survey available research, apply inclusion and exclusion criteria, extract relevant data (with graded evidence levels and risk-of-bias assessments), and synthesize findings, often via meta-analysis, to summarize quantitative evidence. Increasingly, researchers have leveraged these rich textual and quantitative narratives to construct more clinically realistic QA datasets.^8^^,^^9^^,^^10^ Given the near absence of large-scale, human-verified clinical questions, semi-synthetic corpora, in which LLMs transform structured review content into QA pairs, offer a practical and scalable middle ground. They balance the scarcity of expert-annotated data with the potential inaccuracies introduced by automated generation, enabling realistic, up-to-date evaluation of models in evidence-based reasoning.^8^^,^^11^

However, existing QA datasets are primarily designed to serve as benchmarks for LLMs and often fail to examine deeper characteristics of the underlying evidence (e.g., whether the cited studies are well established or frequently cited or the subject matter of the QA pair) and how these characteristics affect model accuracy. Moreover, most evaluations are structured as benchmarks of so-called “zero-shot” ability, where LLMs must answer without access to external tools for literature search or retrieval.

To address these gaps, we construct a comprehensive, multi-source QA dataset to evaluate LLMs’ ability to answer clinical questions and reason over supporting evidence. Our dataset includes questions derived from Cochrane systematic reviews and structured recommendation guidelines from medical associations. With this diverse and carefully curated corpus, we aim to do the following.

(1) Assess the current performance of leading LLMs (as of August 2025, GPT-4o-mini and GPT-5 for small-scale and large-scale all-purpose language models, respectively).
(2) Report associations such as LLMs tending to provide more accurate answers for questions supported by better-cited studies.
(3) Evaluate LLMs in a retrieval-augmented generation (RAG) setting, where models can query PubMed as a proxy for a web search, allowing us to measure how access to external evidence changes accuracy and reasoning.

Biomedical and clinical QA research has been shaped by datasets such as PubMedQA^12^ and BioASQ-QA,^8^ which feature expert-annotated questions grounded in research abstracts. PubMedQA provides yes/no/maybe questions on research articles, while BioASQ-QA includes multi-format questions and summary answers, supporting both factual retrieval and summarization. More recent datasets, such as MIRIAD,^5^ scale to web collections, providing millions of QA pairs to enhance diversity and practical relevance.

HealthFC^3^ examines the alignment between health claims and supporting/refuting evidence, annotated for veracity and strength. CONFLICTINGQA^4^ collects controversial queries with conflicting evidence, showing that LLMs often prioritize surface relevance over deeper reasoning. MedREQAL^9^ introduces QA pairs from Cochrane reviews, emphasizing recall and justification. MedEvidence^10^ directly compares LLM outputs against review conclusions, probing evidence synthesis. Beyond reviews, clinical guidelines provide structured recommendations based on evidence or consensus, and adherence is critical for decision-making.^13^ Recent work has evaluated LLMs against these standards: MedGUIDE^14^ tests adherence to decision trees from guidelines and AMEGA^15^ offers a broad benchmark spanning diagnosis, reasoning, and treatment planning.

Closest to our work are MedEvidence and MedREQAL, both of which build on systematic review datasets. Our work extends these efforts in three key directions. First, we incorporate a diverse set of clinical guidelines, which better capture real-world information flows and clinical decision-making contexts. Second, we provide a granular performance decomposition to identify sources of model strength and failure across publication years, field of medicine, and citation counts. Finally, building on works in prediction-powered inference (PPI) and measurement-error correction frameworks,^16^^,^^17^ we are among the first benchmark-dataset papers to report model performance that explicitly accounts for errors in LLM-generated answers.

## Results

We evaluated a baseline model (GPT-4o-mini) and an advanced model (GPT-5) on three distinct clinical QA tasks. Our findings show that while GPT-5 consistently outperforms the baseline, both models exhibit similar performance patterns. Performance is highest on structured data, varies significantly by evidence prominence and clinical domain, and improves substantially with contextual information.

### Assessing LLM-generated QA alignment with human experts

Across inspected abstracts, human reviewers rated the LLM-generated questions to be appropriately grounded in the corresponding abstracts. Across the reviewer-level counts provided, the pooled human agreement rate was 85% (95% confidence interval [CI]: 77%–91%), with modest between-reviewer dispersion (range: 68%–96%; SD = 8%) that was largely attributable to differences in how a single reviewer operationalized “no” vs. “no evidence” in their answers rather than inherent differences across reviewers.

Reviewer comments converged on a small set of recurring failure modes. First, when generating QA pairs, the LLM occasionally anchored on a single sentence or localized detail, producing questions that were faithful to that fragment but insufficiently reflective of the abstract as a whole. Although the resulting QA pairs were generally valid, reviewers noted that additional prompting or evaluation criteria that encourage more holistic synthesis could be a valuable next step. Second, reviewers observed a preference for confident, scientific-sounding conclusions that sometimes understated uncertainty or overlooked explicitly low or very low levels of evidence, indicating a bias toward certainty over nuance. Third, while *p* values were generally interpreted correctly, LLMs sometimes failed to recognize that a CI for an odds ratio that excludes 1.0 constitutes statistical significance even in the absence of a reported *p* value.

### Performance on systematic reviews

On systematic review abstracts, GPT-5 outperformed GPT-4o-mini across all tasks by a small margin (2%–6%), with the largest gap in overall answer accuracy. Both models showed weaker performance on discrepancy detection and evidence-quality classification (Table 1). Additional confusion matrices are provided in Figure S2. Based on our human reviewer calibration samples, PPI (prediction-powered inference) adjustments only nudged the estimates downwards slightly: for GPT-4o-mini, the uncorrected accuracy is 60.3% (95% CI: 59.3%–61.3%) vs. a PPI of 58.2% (95% CI: 55.0%–61.3%); for GPT-5, the plain accuracy is 67.8% (95% CI: 66.8%–68.8%) vs. a PPI of 65.6% (95% CI 62.5%–68.8%).

**Table 1:** Systematic review performance (GPT-4o-mini vs. GPT-5) with per-class precision/recall/F1

Overall accuracy		
Task	GPT-4o-mini	GPT-5
Answer	60.3% [59.3, 61.3]	67.8% [66.8, 68.8]
Discrepancy	57.0% [55.9, 58.1]	59.1% [58.1, 60.1]
Evidence quality	32.1% [31.1, 33.1]	38.8% [37.8, 39.8]

Answer classification							
Answer	GPT-4o-mini			GPT-5			Support
	Prec.	Rec.	F1	Prec.	Rec.	F1	
No	0.49	0.40	0.44	0.58	0.65	0.61	2,309
No evidence	0.19	0.17	0.18	0.30	0.37	0.33	1,054
Yes	0.71	0.78	0.74	0.84	0.75	0.80	5,167

Discrepancy classification							
Class	GPT-4o-mini			GPT-5			Support
	Prec.	Rec.	F1	Prec.	Rec.	F1	
Missing	0.52	0.17	0.26	0.48	0.51	0.49	2,756
No	0.69	0.78	0.73	0.76	0.64	0.70	5,544
Yes	0.05	0.30	0.09	0.08	0.33	0.13	230

Supports are identical per class across LLMs. Overall accuracy is shown (GPT-4o-mini vs. GPT-5). Prec., precision; Rec., recall. See also Figure S2.

**Figure 1:**
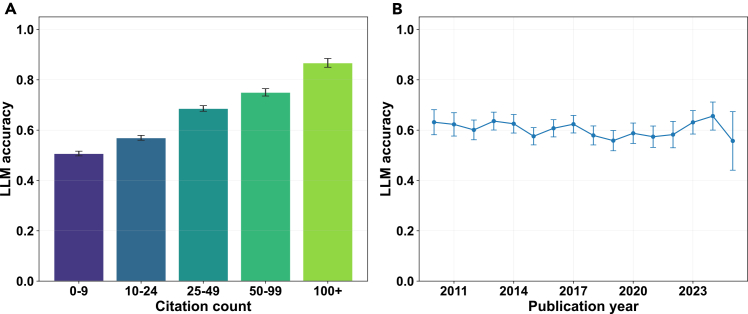
Associations between GPT-4o-mini accuracy and citation impact and publication year We examined associations between GPT-4o-mini accuracy and systematic-review characteristics, including citation impact and publication year. (A) Model answer accuracy by citation count bin with 95% CIs. (B) Model answer accuracy by publication year with 95% CIs. See also Figure S1.

Model accuracy was positively associated with the citation counts of the source review. For GPT-4o-mini, accuracy increased from about 50% for reviews with fewer than 10 citations to nearly 80% for those with more than 100 (*p* < 0.001). GPT-5 showed the same trend, rising from 59.1% in the lowest citation bracket to 79.1% in the highest (odds ratio for log(1+citations)=1.34, 95% CI: 1.29–1.40; see Figure 1). To test whether this effect was simply due to older papers having more time to accumulate citations, we examined accuracy by publication year. Performance remained relatively stable between 2010 and 2015 (55%–65%) and did not increase monotonically with paper age; in fact, there was a slight decline for reviews published in 2025, most likely reflecting the temporal cutoff of model training data (Figure 1). Together, these results suggest that the observed citation effect reflects the prominence and impact of the underlying research rather than the age of the publication.

To assess variability across medical domains, we classified each systematic review into one of 37 primary research areas defined by the Cochrane classification. Performance varied considerably across domains for both models, with no strong correlation between the number of articles published in the primary research area and accuracy (r=−0.14). GPT-4o-mini achieved its highest accuracy in rheumatology (72.7%) and lowest in wounds (43.4%). GPT-5 showed a similar pattern, performing best in tobacco, drugs, and alcohol (74.6%) and worst in health and safety at work (52.1%). Table 2 summarizes the top and bottom five domains for GPT-4o-mini (and corresponding GPT-5 model accuracy).

**Table 2:** Top and bottom 5 topics by answer accuracy (%): GPT-4o-mini vs. GPT-5

Topic	Count	GPT-4o-mini	GPT-5
**Top 5 topics**			

Rheumatology	66	72.7% [62.0, 83.4]	70.8% [59.8, 81.8]
Pain and anesthesia	337	68.8% [63.9, 73.7]	72.1% [67.3, 76.9]
Tobacco, drugs, and alcohol	169	68.6% [61.6, 75.6]	74.6% [68.0, 81.2]
Public health	108	68.5% [59.7, 77.3]	65.7% [56.7, 74.7]
Urology	108	67.6% [58.8, 76.4]	66.7% [57.8, 75.6]

**Bottom 5 topics**			

Health and safety at work	48	54.2% [40.1, 68.3]	52.1% [38.0, 66.2]
Complementary and Alt. Med.	92	53.3% [43.1, 63.5]	57.6% [47.5, 67.7]
Dentistry and oral health	216	53.2% [46.5, 59.9]	61.1% [54.6, 67.6]
Neonatal care	400	50.5% [45.6, 55.4]	68.8% [64.3, 73.3]
Wounds	173	43.4% [36.0, 50.8]	61.8% [54.6, 69.0]

95% CI is shown in brackets. Alt. Med., alternative medicine.

**Figure 2:**
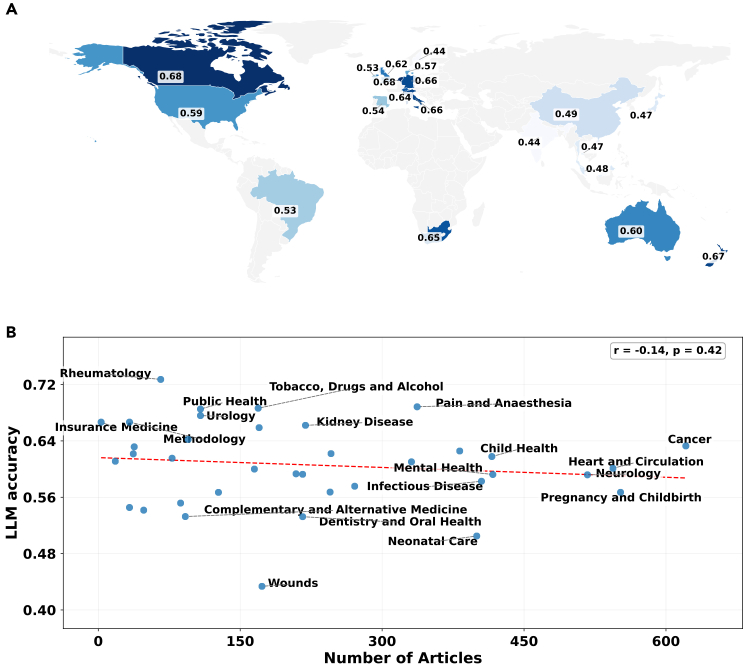
Associations between GPT-4o-mini accuracy and author geography and domain prevalence We examined associations between GPT-4o-mini accuracy and author geography and medical domain prevalence. (A) GPT-4o-mini accuracy on systematic-review questions by majority author-affiliation country (support ≥ 50), shown as a choropleth. (B) GPT-4o-mini accuracy stratified by medical domain vs. number of articles per domain. A regression line is shown (Pearson r=−0.14; p=0.42), confirming no significant association between domain prevalence and model accuracy.

We also assessed whether model accuracy varied by the geographical location of the study team. We mapped each systematic review DOI to its majority author-affiliation country (support ≥ 50) and overlaid GPT-4o-mini accuracy (Figure 2). Accuracy ranges from roughly 0.44 (e.g., India) to 0.68 (e.g., Canada and the Netherlands), with the US, the UK, and Australia clustered around 0.59–0.62 and Germany, Italy, and New Zealand in the mid-0.60s. No monotonic regional pattern emerges, suggesting geography might not be a primary driver of performance.

A detailed error analysis of GPT-4o-mini indicates strong performance on affirmative judgments but persistent weakness on “no evidence” cases. As summarized in Table 1, GPT-4o-mini achieves its highest F1 on “yes” answers (F1 = 0.74; precision = 0.71, recall = 0.78), with notably lower F1 on “no” (0.44) and especially “no evidence” (0.18). For discrepancy detection, GPT-4o-mini shows solid specificity for “no” (F1 = 0.73) but very low sensitivity for “yes” discrepancy (F1 = 0.09; see Table 1). This pattern suggests that the model tends to produce confident, plausible responses and under-calls uncertainty or conflicting evidence. A representative error case is shown in Table 3, where GPT-4o-mini generated a fluent but factually incorrect rationale that contradicted high-quality trial evidence. The consistent overconfidence in “no evidence” cases is consistent with recent analyses that benchmark incentives can favor guessing over abstention in uncertain settings.^18^

**Table 3:** Illustrative error example showing GPT-4o-mini’s wrong reasoning on antiplatelet therapy

Question	do antiplatelet agents reduce all-cause mortality in patients with intermittent claudication compared to placebo?
Model answer	no
Model notes	current evidence … suggests that they do not significantly reduce all-cause mortality …
Ground truth	yes
Notes	antiplatelet agents reduced all-cause mortality with a risk ratio of 0.76 (95% CI: 0.60–0.98)

**Figure 3:**
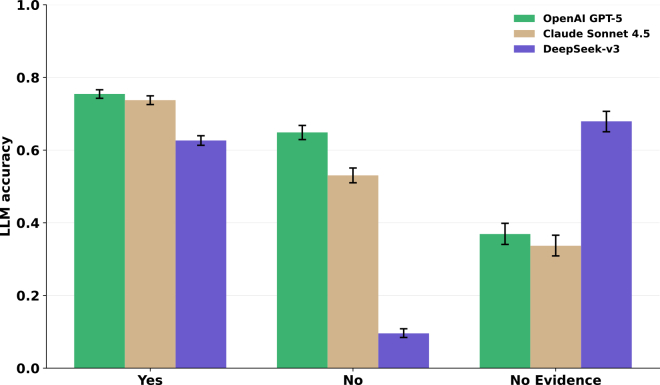
Comparison of accuracy by answer type across GPT-5, Claude 4.5, and DeepSeek-v3 Comparison of accuracy by answer type across GPT-5, Claude 4.5, and DeepSeek-v3 with Wilson 95% confidence intervals. Patterns remain consistent across major answer categories.

To assess whether the observed patterns generalize beyond the ChatGPT family, we replicated all experiments using Claude 4.5 Sonnet and DeepSeek-v3. A comparison of answer accuracy is presented in Figure 3. Inter-model concordance was high for Cochrane questions (N=8,530): GPT-5 vs. Claude 4.5 achieved 75.0% raw agreement (Cohen’s κ=0.57; Pearson r=0.62; p<0.001), and GPT-5 vs. DeepSeek-v3 achieved 54.4% agreement (Cohen’s κ=0.31; Pearson r=0.48; p<0.001). Additional cross-model plots (citations, year, and clinical field) are provided in Figure S1.

### Performance on structured clinical guidelines

**Figure 4:**
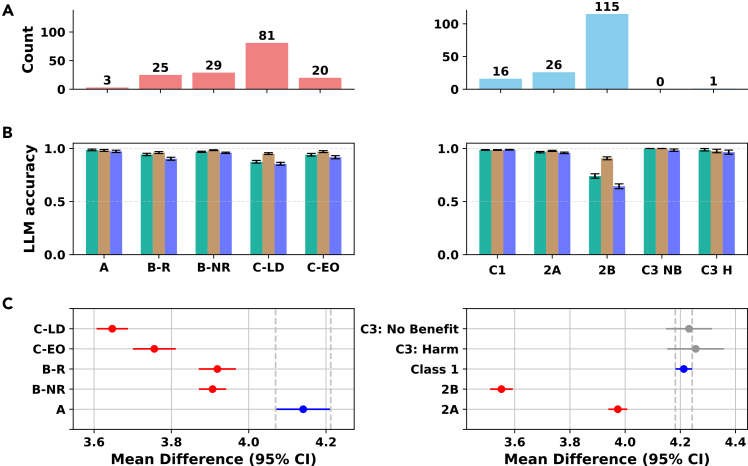
AHA guideline results The images summarize error distributions, cross-model accuracy, and model score alignment with LOE/COR hierarchies. Each row presents paired panels: LOE categories (left) and COR categories (right). (A) Distribution of model-incorrect cases across guideline-defined evidence levels (LOE, left) and recommendation strengths (COR, right). Errors cluster in weak- or low-confidence categories (C-LD, C-EO, and class 2B). (B) Cross-model accuracy by evidence category: LOE (left) and COR (right) with binomial standard error bars. Models show consistent patterns across categories. (C) Tukey HSD analysis of model-assigned scores vs. ground-truth evidence levels (LOE, left) and recommendation strengths (COR, right). Higher ratings correspond to stronger evidence and recommendations, while weaker categories (C-LD, C-EO, and class 2B) received the lowest scores.

When evaluated on highly structured recommendations from AHA (American Heart Association) guidelines, both models performed exceptionally well. GPT-4o-mini achieved 94.0% accuracy (precision = 1.00, recall = 0.94, and $F_{1}=0.97$, with 158 incorrect predictions). Importantly, the errors were concentrated in cases where the guidelines themselves provided weaker evidentiary support. Specifically, incorrect predictions clustered in recommendations with level of evidence (LOE) C-LD (limited data) or C-EO (expert opinion) and in class of recommendation (COR) 2B, which represents weak positive recommendations. In contrast, errors were rare for recommendations backed by strong trial evidence (LOE A) or those classified as unequivocal benefit or harm (classes 1 and 3). This indicates that the model’s uncertainty mirrors the ambiguity present in clinical evidence. We display this observation in Figure 4A: the majority of misclassifications occur in LOE C-LD (81 cases) and C-EO (20 cases), compared to very few in LOE A (3 cases), and nearly all COR-related errors fall in class 2B (115 cases), with almost none in classes 1 and 3.

Beyond raw accuracy, we examined whether the model’s assigned evidence-quality and recommendation-strength scores aligned with the clinical hierarchy. Figure 4C shows the Tukey HSD (honestly significant difference) analysis comparing model-assigned scores against guideline-defined categories: LOE A receives the highest scores (around 4.1–4.2), B-R (level B, recommendation sourced from one or more well-designed randomized control trials) and B-NR (level B, moderate evidence from well-designed non-randomized trials) intermediate (around 3.9), and C-LD (level C, based on limited or low-quality studies)/C-EO (level C, based on expert opinions when research data is sparse) the lowest (3.6–3.8), with classes 1 and 3 rated highest for strength, 2A slightly lower, and 2B the lowest (around 3.6).

On this task, Claude 4.5 and DeepSeek-v3 achieve overall accuracies of 97.0% and 91.9%, respectively, compared with the GPT-4o-mini baseline at 93.8%. Claude 4.5 vs. DeepSeek-chat show 92.0% raw agreement (Cohen’s κ=0.25; Pearson r=0.22; p<0.001). Figure 4B presents cross-model accuracy by LOE (top) and COR (bottom), showing consistent patterns across GPT-4o-mini, Claude 4.5, and DeepSeek-chat.

### Performance on narrative clinical guidelines

In contrast to the structured AHA task, model accuracy dropped significantly on questions from unstructured, narrative text. GPT-4o-mini’s accuracy fell to 56.3%, where it particularly failed to correctly interpret statements of negative findings (31.6% accuracy on “no” answers). This issue was especially pronounced for sentences containing double negations or complex phrases indicating a lack of efficacy. For instance, when asked whether dry needling combined with guideline-based physical therapy provides additional benefit in patients with chronic neck pain, the GPT model incorrectly answered “yes” despite the source text explicitly stated the intervention “provides no added benefit,” demonstrating a bias toward affirmative responses when faced with nuanced negative language. We also report class-wise precision/recall/F1 for GPT-4o-mini on the narrative set (N=10,456) in Table 4, showing high metrics on “yes” but low metrics for “no evidence.” To validate the robustness of the findings, we tested the same questions on two additional models (Claude 4.5 and DeepSeek-v3). For narrative guideline questions (N=10,456), Claude 4.5 vs. DeepSeek-v3 showed 66.8% agreement (Cohen’s κ=0.434; Pearson r=0.557; p<0.001), indicating broadly consistent behavior across frontier models with greater variance for the open-weight system.

**Table 4:** Narrative guideline error example and classification metrics

Example of a model error on a narrative guideline QA	
Question	in patients with chronic neck pain, does dry needling combined with guideline-based physical therapy provide additional benefit?
Ground truth	no
Model answer	yes
Supporting evidence	dry needling combined with guideline-based physical therapy provides no added benefit …

Answer	Precision	Recall	F1-score	Support
**Classification report (GPT-4o-mini) on narrative guideline answers**				

No	0.46	0.32	0.37	1450
No evidence	0.05	0.61	0.09	316
Yes	0.94	0.60	0.73	8,690

Table 5 shows that model errors are asymmetric in quantity. One dominant failure mode is hedging in the presence of evidence, where questions with established benefit or harm are predicted as “no evidence” (e.g., yes → no evidence or no → no evidence), especially for narrative guidelines. Another major pattern is overassertion of benefit under weak or insufficient evidence, where “no evidence” or negative findings are incorrectly predicted as “yes.” Finally, models also exhibit missed benefits, predicting “no” for supported interventions, often reflecting over-skepticism or reliance on outdated or weak signals.

**Table 5:** Error modes with counts and representative failures across sources

Ground truth	Prediction	Count	Error type	Example question (representative failure)	Source
No evidence	yes	622	over-asserted benefit	do pharmacological interventions improve anxiety symptoms in COPD? (evidence insufficient; predicted yes)	systematic review
Yes	no	706	missed benefit/dated knowledge	do non-aspirin NSAIDs reduce Parkinson’s risk? (protective signal down-weighted; predicted no)	systematic review
No	yes	1,035	overgeneralization from weak signals	is fluoxetine more effective than placebo for seasonal affective disorder? (Relative Risk nonsignificant; predicted yes)	systematic review
Yes	no evidence	420	hedging negative conclusion	does naftidrofuryl improve function in dementia? (reported benefit; predicted no evidence)	systematic review
No evidence	no	251	conservative abstention	does lycopene reduce prostate cancer incidence? (evidence insufficient; predicted no)	systematic review
No	no evidence	349	hedging negative conclusion	are cholinesterase inhibitors effective for delirium duration in non-ICU? (negative MD; predicted no evidence)	systematic review
No evidence	yes	117	over-asserted benefit	in individuals with asthma, does carpet removal improve asthma outcomes? (insufficient evidence; predicted yes)	narrative guideline
Yes	no	534	missed benefit/skeptical	sofosbuvir/velpatasvir/voxilaprevir 8-week regimen for HCV genotype 3 with cirrhosis achieving 96% SVR12? (supported; predicted no)	narrative guideline
No	yes	241	overgeneralization from weak signals	do impermeable pillows reduce asthma attacks vs. placebo pillows? (negative; predicted yes)	narrative guideline
Yes	no evidence	2,923	hedging negative conclusion	is an intervention acceptable due to minimal harm? (supported; predicted no evidence)	narrative guideline
No evidence	no	6	conservative abstention	is TMP-SMX safe in pregnant women? (insufficient direct evidence; predicted no)	narrative guideline
No	no evidence	751	hedging negative conclusion	do impermeable bedding covers improve QoL in children with asthma? (negative; predicted no evidence)	narrative guideline
Yes	no	26	missed benefit/contradicts guideline	effectiveness of tranexamic acid for spontaneous intracerebral hemorrhage deemed “not well established” (predicted as no)	AHA guideline
Yes	unknown	132	hedging/uncertainty	PCI to improve survival in SIHD with three-vessel disease predicted as no evidence/unknown despite conditional support	AHA guideline

### Impact of RAG

To evaluate whether external context improves model performance, we re-ran the systematic review tasks under different contextual conditions. We evaluated three context configurations designed to probe complementary aspects of model behavior. First, we provided the gold-source abstract corresponding to the originating systematic review, which serves as an approximate upper bound on performance when perfectly relevant evidence is available. Second, we supplied a randomly sampled abstract unrelated to the question as a negative control to assess robustness to irrelevant or noisy context. Third, we used the top three abstracts returned by PubMed relevance-ranked retrieval, reflecting a realistic end-to-end deployment scenario in which evidence is retrieved automatically rather than curated. In all cases, retrieved text was appended directly to the prompt as background context, and PubMed results were taken from the highest-ranked hits returned by the search interface. As summarized in Table 6, providing the correct source abstract increased accuracy for both models, surpassing 90%. A more realistic setup using PubMed-retrieved abstracts also yielded substantial gains, boosting GPT-4o-mini from a baseline of 60.3% to 79.9% and GPT-5 from 67.8% to 75.2% on the tested subset. In contrast, irrelevant context (random abstracts) produced only a slight degradation in accuracy, suggesting both models are relatively robust to noisy inputs. These results highlight retrieval-augmented prompting as a practical and effective strategy for improving performance on systematic review-based clinical QA.

**Table 6:** Model accuracy on abstract-based QA with different contexts

Context condition	GPT-4o-mini	GPT-5^a^
No context (baseline)	60.3% [56.0, 64.6]	67.8% [63.7, 71.9]
Correct abstract (Oracle)	91.6% [89.2, 94.0]	93.2% [91.0, 95.4]
PubMed retrieval	79.9% [76.4, 83.4]	75.2% [71.4, 79.0]
Random abstract (noise)	58.1% [53.8, 62.4]	65.1% [60.9, 69.3]

a GPT-5 contextual results extrapolated from a 500-case subset of previously incorrect answers.

### Qualitative analysis of model output

Beyond classification metrics, we also inspected LLM’s explanations for selected correct and incorrect predictions to understand how the model justified its answers. We found that GPT-5’s outputs reveal context-aware reasoning that distinguishes study designs, recognizes low-quality or limited evidence, and flags mixed findings—often resembling the logic of a systematic reviewer. For instance, when asked whether erythropoiesis-stimulating agents improve exercise capacity,^19^ GPT-5 correctly noted that large randomized controlled trials (RCTs) found no improvement in 6-min walk distance but did report increased exercise duration, mirroring the review’s nuanced interpretation.

We observed several failure modes: first, the model frequently inflated evidence strength, overstating conclusions. For example, when asked whether scapular fixation in muscular dystrophy improves upper-limb function, GPT-5 acknowledged that no randomized trials exist and all data were observational, yet it still predicted “yes” instead of “no evidence.”^20^ Second, the model sometimes over-relied on lexical cues such as “no significant,” misclassifying positive quantitative results as negative. Third, it occasionally conflated “no” (confirmed lack of benefit) with “no evidence” (absence of statistically significant findings), as in Christie et al.,^21^ where an RCT comparing cemented vs. uncemented humeral stem fixation showed nonsignificant differences but GPT-5 answered “no.”

Despite these discrepancies, GPT-5’s reasoning broadly reflected human-style evidence synthesis, often using phrases such as “limited evidence” or “mixed results.” Incorrect cases disproportionately contained such hedging, suggesting that linguistic uncertainty correlates with factual error. This parallels recent work showing that LLMs exhibit human-like but miscalibrated uncertainty behavior.^22^^,^^23^

### Comparing human- vs. LLM-generated questions

**Figure 5:**
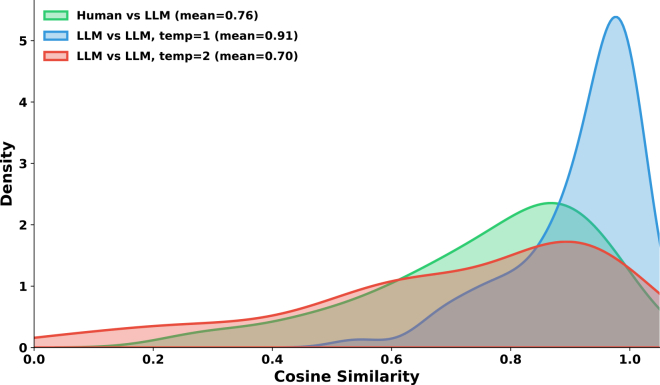
Distribution of cosine similarity for questions generated from the same abstract Human-vs.-LLM similarity (green) falls between LLM-vs.-LLM similarity at temperature 1 (blue, high consistency) and temperature 2 (red, high variability), indicating that human questions diverge from LLM questions about as much as high-temperature LLM outputs diverge from each other.

To quantify the difference between human- and LLM-generated questions, we computed cosine similarity between sentence embeddings^24^ (all-MiniLM-L6-v2) for questions generated from the same abstract. To contextualize the resulting similarity, we also vary the variability of LLM generation using the temperature parameter *T* for GPT-4o and generated pairs of questions for the same abstract at T=1 and T=2. The results (Figure 5) show that human-vs.-LLM similarity (mean = 0.76) falls between LLM-vs.-LLM similarity at temperature 1 (mean = 0.91) and temperature 2 (mean = 0.70). In other words, human questions differ from LLM questions roughly as much as two LLM outputs at high temperature differ from each other.

Upon review, human reviewers frequently adopted an evidence-seeking stance, asking whether trials exist, whether evidence is sufficient, or whether any conclusion can be drawn at all (e.g., “Are there RCTs that evaluated … ?” and “Is there evidence that … ?”). By contrast, LLM-generated questions follow a more uniform population-intervention-comparison-outcome-style template (e.g., “In population P, does X reduce Y compared to Z?”), presupposing that relevant evidence exists. Human questions also tend toward broader phrasing (“more benefits” and “establish optimal treatment”), while LLM questions anchor to specific outcomes and studies.

This distribution shift degrades model accuracy, consistent with growing evidence that LLMs tend to favor their own generations and perform better on questions generated by other LLMs.^25^^,^^26^ Across the 75 human-authored questions, GPT-5 achieved 48.7% accuracy (95% CI: 37.8%–59.7%), compared to 42.1% for Claude Sonnet, 38.2% for GPT-4o-mini, and 31.6% for DeepSeek—roughly 10 percentage points lower than on LLM-generated questions. The model ranking remains consistent, suggesting that while LLM-generated benchmarks overestimate absolute performance, they still provide a useful signal for comparing models.

## Discussion

We present a new dataset and accompanying in-depth analysis for evaluating LLMs in evidence-based clinical QA, grounded in high-quality sources including systematic reviews and clinical guidelines. Compared with existing biomedical QA datasets^8^^,^^12^ and more recent large-scale collections,^5^ our dataset emphasizes reasoning about evidence quality, recommendation strength, and discrepancies between study designs. This focus aligns with recent calls for benchmark tasks that move beyond simple fact retrieval toward assessing how models handle conflicting or uncertain evidence.^3^^,^^4^

Our QA generation parallels human evidence assessment in evidence-based medicine: studies are weighted by methodological rigor and bias risk within hierarchies of evidence. By grounding QA in Cochrane reviews and guideline frameworks (AHA COR/LOE), the task mirrors how clinicians synthesize and weigh evidence strength and reliability when forming judgments. Looking ahead, such evidence-aware QA systems could be integrated into clinical workflows as intelligent companions within electronic health records or clinical decision support systems.^27^^,^^28^ Achieving this will require rigorous attention to model transparency, citation provenance, and uncertainty calibration, as well as human-in-the-loop feedback mechanisms to ensure that AI-generated answers meaningfully augment clinician reasoning.^29^^,^^30^

Our evaluation reveals several key findings. First, base models achieve only moderate accuracy on systematic-review-derived questions, reflecting common error modes such as gaps in domain-specific knowledge and failures when evidence is absent or ambiguous. Second, performance is markedly higher on structured recommendations from clinical guidelines, consistent with prior observations that LLMs excel on templated biomedical tasks.^31^ Third, even without explicit context, models show some ability to differentiate the underlying evidence quality and recommendation strength of unstructured guideline recommendations. By contrast, evidence quality is much harder to recover from free-text systematic reviews or narrative guideline statements, where double negation and hedging language likely introduce substantial error. Fourth, performance varies systematically by domain and citation impact of the source literature, suggesting that model predictions are influenced not only by input complexity but also by the external visibility of the underlying evidence. Finally, in-context learning and retrieval-augmented prompting substantially improve performance, consistent with broader findings in biomedical RAG.^32^^,^^33^

### Limitations of the study

Our findings should be interpreted in light of several limitations. First, a substantial fraction of the QA pairs in our benchmark were automatically generated using LLMs. Although manual spot checks indicated high consistency with the source documents, residual errors and ambiguities may remain. More systematic adoption of calibration-based approaches, such as PPI,^16^ will be important in future work to enable more principled uncertainty quantification. Second, while our data sources are high quality, they are not exhaustive. By focusing on Cochrane systematic reviews and major clinical guidelines, we necessarily excluded other specialties and forms of gray literature, which may contain emerging or preliminary evidence that has not yet been synthesized in formal reviews. Given our emphasis on established, rigorously vetted evidence, this represents a deliberate trade-off between timeliness and methodological rigor. Third, our answer formats were largely restricted to categorical labels to facilitate scalable and reliable evaluation. While this design choice improves comparability and efficiency, it may oversimplify complex clinical reasoning processes and preclude more nuanced justifications. In particular, medical reasoning often requires contextual explanation (e.g., aggregated effect sizes from different studies and subgroup effects) that cannot be fully captured by discrete labels. As the AI-for-medicine community increasingly invests in benchmarks with open-ended responses,^34^^,^^35^ future work may leverage these advances to evaluate and grade free-text model outputs.

Future work may extend our proposed benchmark along several complementary dimensions, including incorporating additional evidence sources, supporting richer and more structured answer formats, and expanding the role of human expert validation, including investigating efficient strategies for sampling generated QA pairs for human labeling. In parallel, integrating LLM-based QA with retrieval pipelines over high-quality biomedical databases and clinical guideline repositories offers a promising path toward safer and more robust clinical decision support systems. Moreover, future studies could explicitly incorporate a temporal dimension by evaluating whether LLMs can accurately anticipate the conclusions of newly published systematic reviews that appear after the model’s knowledge cutoff. Finally, testing LLMs’ ability to answer complex, nuanced medical questions in languages other than English would further our understanding of their global generalizability.

As LLMs continue to be deployed in clinical and public health contexts, we hope that our proposed benchmark and findings will help inform model development, evaluation practices, and performance analysis in evidence-based medical applications.

## Methods

### Dataset construction

We constructed a multi-source clinical QA dataset from three evidence streams: Cochrane systematic reviews, AHA guideline recommendations, and narrative clinical guidelines. The Cochrane dataset was obtained from https://www.cochranelibrary.com/ and comprised 8,533 abstracts of completed reviews published between 2010 and May 2025 (excluding protocols), along with associated metadata (DOI, PubMed ID, title, abstract, authors, affiliations, year, and citation counts). The AHA guidelines dataset comprised 2,581 structured recommendations issued between 2020 and 2025, each annotated with a normalized COR and LOE (available at https://professional.heart.org/en/guidelines-statements-search). Narrative guidelines consisted of 289 documents drawn from US professional societies and major insurers.

Cochrane abstracts were enumerated via PubMed and Cochrane Library DOIs; metadata and structured abstracts were retrieved programmatically. For the AHA guidelines, recommendations were extracted from machine-readable tables, appendices, and inline text. Narrative guidelines were collected from full-text or compiled sources, with duplicates and non-guideline content removed.

For dataset generation, GPT-4o produced structured questions and answers across all sources. From Cochrane abstracts, we derived three types of outputs: (1) a clinically relevant question with one of ({yes, no, no evidence}), (2) a question on whether the abstract reported discrepancies between findings from observational studies and RCTs, and (3) a label for the overall quality of evidence, constrained to categorical values (five different levels).

For AHA recommendations, we generated (1) a judgment of whether the recommendation was supported by evidence ({yes, no, unknown}), (2) a rating of perceived recommendation strength, and (3) a rating of evidence quality. These labels were mapped directly to the AHA’s guideline framework, which encodes both the strength and certainty of evidence. Specifically, the COR indicates the strength of a clinical recommendation and has four categories: class I (strong), class IIa (moderate), class IIb (weak), and class III (no benefit or harm). The LOE indicates the type and quality of supporting evidence, with three main categories: level A (high-quality evidence from multiple randomized trials or meta-analyses), level B (moderate-quality evidence, including single randomized trials or nonrandomized studies), and level C (expert opinion or limited data).

Finally, for narrative clinical guidelines, we sourced documents from leading insurance companies in the US, which regularly publish updated clinical guidance for clinicians across a wide range of medical specialties. Because clinical guidelines vary substantially in length (ranging from as few as 3 to over 100 pages in our collected samples), we standardized the input by segmenting each document into contiguous chunks of approximately 2,000 characters. From each chunk, GPT-4o generated a structured clinical question specifying the population, intervention (or exposure), comparator, and outcome. Model answers were constrained to the categorical set {yes, no, no evidence}. Across 289 guidelines, this chunking procedure produced 13,290 contiguous slices (median 14 slices per guideline, interquartile range [IQR]: 6–43; 90th percentile ≈ 102; 95th percentile ≈ 151). Representative QA pairs are provided in the supplemental information.

To ensure quality and comparability, we harmonized COR/LOE categories across sources and required rationales to directly quote or closely paraphrase source text. Duplicate records were removed. Overall, our curation process yielded a total of approximately 21,000 QA pairs across sources (8,530 from Cochrane systematic review abstracts, 2,580 from AHA structured recommendations, and 10,500 from narrative clinical guideline documents). Table 7 shows representative examples from each data source.

**Table 7:** Examples of generated questions and annotations across three data sources

Cochrane review abstract	
Title	chemoradiotherapy for cervical cancer: meta-analysis
Question	does chemoradiotherapy improve 5-year survival compared with radiotherapy alone in women with cervical cancer?
Answer	yes
Evidence level	high
Discrepancy	no
Note	6% improvement in 5-year survival (Hazard Ratio = 0.81, p<0.001).
AHA guideline recommendation	
Recommendation	for intermediate-risk patients with acute chest pain and no known coronary artery disease, a rest-stress myocardial perfusion imaging study is reasonable
Questions	Q1: supported by evidence? Q2: strength (1–5)? Q3: evidence quality (1–5)?
Labels	class of recommendation (COR): IIa; level of evidence (LOE): A
Source	Gulati et al.,^36^ AHA/ACC Chest Pain Guideline
Narrative clinical guideline	
Question	in adults with chronic heart failure, does exercise therapy improve quality of life?
Answer	yes
Snippet	reports improved quality of life scores and physical capacity with structured exercise therapy

Because automated extraction by LLMs does not always capture the nuances of evidence interpretation and because even the best-performing LLMs can hallucinate, we conducted manual verification on subsamples of QA pairs derived from both Cochrane systematic reviews and narrative clinical guidelines. Five independent reviewers validated 100, 20, 20, 20, and 20 sets of questions, respectively. All reviewers hold a bachelor’s degree in a quantitative science, are currently enrolled in graduate programs in biostatistics or epidemiology, and have extensive experience evaluating real-world medical evidence.

In addition, because LLMs are known to preferentially align with content generated by other LLMs rather than by humans,^25^^,^^26^ we assessed two related questions: first, how similar LLM-generated and human-generated questions are when conditioned on the same abstract; and second, whether model accuracy differs between LLM-generated and human-generated questions. To this end, three reviewers independently generated clinical questions with categorical answers ({yes, no, no evidence}) and corresponding evidence-strength ratings for 25 abstracts. This human-generated corpus was used to compare semantic similarity between LLM- and human-authored questions, using cosine similarity and qualitative comparisons, and to assess differences in downstream answer accuracy.

### Evaluation

We framed clinical QA as a three-way classification task with constrained label sets. To ensure consistency, models were prompted to produce structured outputs with a fixed set of fields (question, answer, evidence-quality, discrepancy, and notes), each restricted to predefined values. The full templates used are provided in the supplemental information.

We evaluated both GPT-4o-mini and GPT-5 to compare performance trends between smaller, fast-inference models and larger frontier reasoning models. Evaluation settings included (1) a no-context baseline (question only), with additional error analysis on a subsample of misclassified questions (performed for two of the three datasets), and (2) context ablations on a challenging subset where the baseline failed, tested under four conditions—no context, the correct abstract, a random abstract, or PubMed top 3 (up to three retrieved abstracts concatenated with separators and excluding the original article, mimicking a realistic retrieval setting). In addition to categorical labels, we also prompted the models to generate brief free-text rationales, enabling probing of model reasoning. To assess the robustness of our findings across LLM families, we compared our results against additional runs using two non-OpenAI models (Claude 4.5 Sonnet and DeepSeek-v3).

### Statistical analysis and reporting

Our primary evaluation metric was exact-match accuracy, defined as the proportion of predictions that exactly matched the ground-truth answer out of all evaluated items. Invalid or out-of-vocabulary outputs were retained in the denominator and scored as incorrect. In addition to accuracy, we reported confusion matrices and class-wise precision, recall, and F1 for the answer, evidence-quality, and discrepancy labels to account for class imbalance. We further conducted analyses to assess associations between model accuracy and clinical field, publication year, citation count, and the primary geographical location of the research team. Unless stated otherwise, all point estimates are reported with 95% CIs constructed using normal approximations.

Because the questioning answer pairs in our benchmark were generated using LLMs, special care is required to account for potential errors in the generated ground-truth answers and their impact on downstream accuracy estimation and inference. As a sensitivity analysis, we calibrated our primary outcome, model answer accuracy, using a PPI framework.^16^ The methodological details are relegated to the supplemental information; the key insight is that the calibration step corrects for systematic errors in the LLM-generated ground truth, enabling valid inference even when most items lack human labels.

## Resource availability

### Lead contact

Requests for further information and resources should be directed to and will be fulfilled by the lead contact, Yiqun Chen (yiqunc@jhu.edu).

### Materials availability

This study did not generate new physical materials.

### Data and code availability

• The curated QA benchmark dataset generated during this study is publicly available and archived on Zenodo (DOI: https://doi.org/10.5281/zenodo.18363151)^37^ and can also be accessed via Hugging Face at https://huggingface.co/datasets/cwang271/MEDAL. All other data reported in this paper will be shared by the lead contact upon request.
• All data processing scripts and evaluation code are available at https://github.com/yiqunchen/MEDAL (also deposited to https://doi.org/10.5281/zenodo.18372374)^38^ and are publicly available as of the date of publication.
• Any additional information required to reanalyze the data reported in this paper is available from the lead contact upon request.

## Acknowledgments

Y.C. receives support from the Johns Hopkins Bloomberg School of Public Health, Department of Biostatistics, Data Science, and the AI Faculty Innovation Fund. We thank Daniel Byrne, Ian Saldanha, Yinuo Tu, Alyssa Columbus, Ding Ding, Sheryl Sun, and Daniel E. Ford for helpful conversations.

## Author contributions

C.W. and Y.C. jointly contributed to data curation, experimental design, analysis, and interpretation of the results. Both authors contributed to writing and revising the manuscript and approved the final version.

## Declaration of interests

The authors declare no competing interests.

## Declaration of generative AI and AI-assisted technologies in the writing process

During the preparation of this work, the authors used ChatGPT for language polishing. After using this tool, the authors reviewed and edited the content as needed and take full responsibility for the content of the publication.

## Supplemental information

Document S1. Figure S1 and supplemental methods

Document S2. Article plus supplemental information
