# Supplementary material for: Evaluating large language models for evidence-based clinical question answering
Source: Patterns (N Y). 2026 Mar 30;7(5):101519. doi: 10.1016/j.patter.2026.101519 (PMC13161685; doi:10.1016/j.patter.2026.101519)
Supplement: Document S1. Figure S1 and — supplemental methods [file mmc1.pdf]

Patterns, Volume *7*

Supplemental information

Evaluating large language models for evidence-based clinical question answering

Can Wang and Yiqun Chen

Supplemental Methods

1. Narrative Guideline Slice Examples

Slice → Question Example 1

Slice (periodicity_schedule_0_slice_2):
1. If a child comes under care for the first time at any point on the schedule, or if any items are not accomplished at the suggested age, the schedule should be brought up to date at the earliest possible time.

2. A prenatal visit is recommended for parents who are at high risk, for first-time parents, and for those who request a conference. The prenatal visit should include anticipatory guidance, pertinent medical history, and a discussion of benefits of breastfeeding and planned method of feeding.

3. Newborns should have an evaluation after birth, and breastfeeding should be encouraged (and instruction and support should be offered).

4. Newborns should have an evaluation within 3 to 5 days of birth and within 48 to 72 hours after discharge from the hospital to include evaluation for feeding and jaundice. Breastfeeding newborns should receive formal breastfeeding evaluation, and their mothers should receive encouragement and instruction. Newborns discharged less than 48 hours after delivery must be examined within 48 hours of discharge.
Generated QA: Should newborns discharged less than 48 hours after delivery be evaluated again within 48–72 hours? (Answer: Yes)

Slice → Question Example 2

Slice (Fever%20in%20Infants__8__slice__2):
Do not use ceftriaxone in hyperbilirubinemic neonates, particularly those who are premature since ceftriaxone is reported to displace bilirubin from albumin binding sites; concomitant use with intravenous calcium-containing solutions/products in neonates $\leq$ 28 days of age is contraindicated.

Indications for HSV Evaluation: ill appearance, hypothermia, seizures, vesicles, mucous membrane ulcers, hepatosplenomegaly, maternal history of genital HSV lesions or concern for primary HSV infection, thrombocytopenia, CSF pleocytosis without a positive Gram stain, elevated ALT.

HSV Work-up: CSF HSV PCR; blood HSV PCR; HSV PCR from conjunctivae, nasopharynx, mouth, and anus; HSV PCR from any suspicious mucocutaneous lesions; ALT.

CSF Studies: cell count and differential; protein; glucose; Gram stain and culture; meningitis/encephalitis PCR panel (send if pleocytosis present: CSF WBC $\geq 18/mm^3$ for age $\leq$ 28 days and CSF WBC $\geq 10/mm^3$ for age 29–60 days).

Decisions on repeat LP should consider source of infection, blood culture results, and clinical presentation.

Process for IR-guided LPs: M–F 7a–5p page IR consult; after hours page IR attending; consider ultrasound to evaluate for hematoma.

Would consider CSF uninterpretable with CSF RBC $\geq$ 10,000 cells/mm^3; interpret CSF WBC at face value for CSF RBC $<$ 10,000 cells/mm^3.

Back to 8–21 days; Back to 22–28 days; Back to 29–60 days
Generated QA: In hyperbilirubinemic neonates, does ceftriaxone increase the risk of bilirubin displacement from albumin binding sites? (Answer: Yes)

2. Prompt Templates

This appendix lists the exact prompts used in the evaluation. Placeholders such as `{question}` are programmatically substituted. For context runs, retrieved abstracts are inserted verbatim under the "Background context" header. When multiple PubMed abstracts are present, they are concatenated with a separator line `--`.

2.1. No-Context Prompt

You are a clinical research expert with knowledge of systematic reviews, RCTs, and observational studies. Task: Given a clinical question, return a JSON with keys question, answer, evidence-quality, discrepancy, notes.
Allowed values: - answer: Yes | No | No Evidence - evidence-quality: High | Moderate | Low | Very Low | Missing - discrepancy: Yes | No | Missing
Question: """question"""

2.2. Context Prompt (scripts/evaluate_with_context.py)

> You are a clinical research expert with knowledge of systematic reviews, RCTs, and observational studies. Task: Given a clinical question, optionally with background abstracts, return a JSON with keys question, answer, evidence-quality, discrepancy, notes.
> Allowed values: - answer: Yes | No | No Evidence - evidence-quality: High | Moderate | Low | Very Low | Missing - discrepancy: Yes | No | Missing
> Background context (may be empty; if multiple abstracts, separate with a line containing only —):
> """context"""
> Question: """question"""

2.3. Notes on Inference Configuration

- Temperature set to `0.2` for non-reasoning models; for `GPT-5` we use `temperature=None` and, when supported, `reasoning_effort=medium`.
- Responses requested as strict JSON via `response_format={type=json_object}` when available; outputs are parsed with JSON deserialization. Invalid outputs are recorded as errors and counted as incorrect.

3. Additional Results with Non-OpenAI Models

To check whether our main findings generalize beyond OpenAI models, we repeated the key analyses with Claude 4.5 and DeepSeek-v3. The same high-level patterns hold: accuracy increases with citation count, shows no clear trend by publication year, and varies substantially by clinical subfield. GPT-5 and Claude 4.5 perform similarly overall, while DeepSeek-v3 is more variable, especially in medical domains with fewer studies.

3.1. Additional Diagnostics Across Models

To complement overall accuracy, we report confusion matrices for GPT-4o-mini on answer prediction and discrepancy detection (Figure S2). Errors concentrate in rarer or more ambiguous classes, most notably "No evidence" answers and "Yes" discrepancy cases. We also found little association between accuracy and the number of papers included per systematic review (Figure S1(c)–(d)).

4. Methodological Details for PPI-based Calibration

We formulate the calibration problem as follows: For each generated question $i = 1, \dots, N$, we observe a triplet $(Y_i, Z_i, \hat{Y}_i)$, where Y_i denotes the (unobserved) true answer that a human expert would provide, Z_i denotes the LLM-generated reference answer produced from the abstract and supporting statements, and $\hat{Y}_i$ denotes the model-generated answer to question i.

The estimand of interest is the true accuracy with respect to human judgment $\mathbb{E}\Big[\mathbf{1}\{Y_i = \hat{Y}_i\}\Big]$, where $\mathbf{1}\{\cdot\}$ denotes the indicator function. However, human-verified answers Y_i are available only for a small calibration subset of size $n \ll N$ (for this work, n is around 200 and N is over 8,000). To obtain unbiased and efficient estimates of the accuracy θ, we exploit the decomposition $\mathbb{E}\Big[\mathbf{1}\{Y_i = \hat{Y}_i\}\Big] = \mathbb{E}\Big[\mathbf{1}\{Y_i = \hat{Y}_i\} - \mathbf{1}\{Z_i = \hat{Y}_i\}\Big] + \mathbb{E}\Big[\mathbf{1}\{Z_i = \hat{Y}_i\}\Big]$, which separates the target accuracy into a correction term that depends on human labels and a large-sample term computable using LLM-generated answers alone.

In practice, we estimate θ by $\hat{\theta}_{\text{PPI}} = \frac{1}{n}\sum_{i=1}^{n}\Big[\mathbf{1}\{Y_i = \hat{Y}_i\} - \mathbf{1}\{Z_i = \hat{Y}_i\}\Big] + \frac{1}{N-n}\sum_{i=n+1}^{N}\mathbf{1}\{Z_i = \hat{Y}_i\}$, where the first term estimates the discrepancy between human-verified and LLM-generated accuracy on

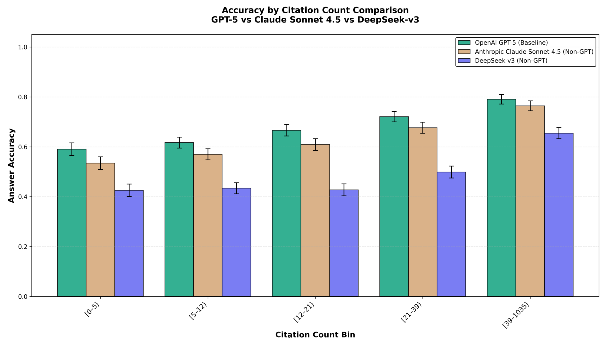

(a) Accuracy by citation count

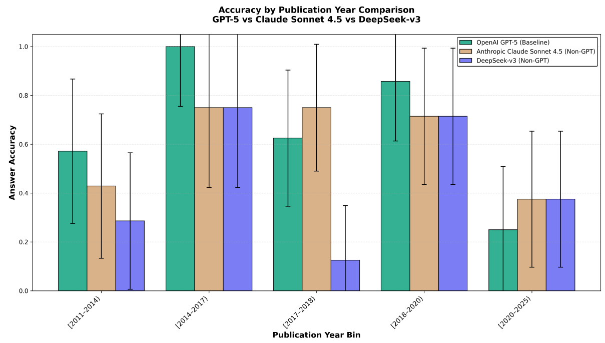

(b) Accuracy by publication year

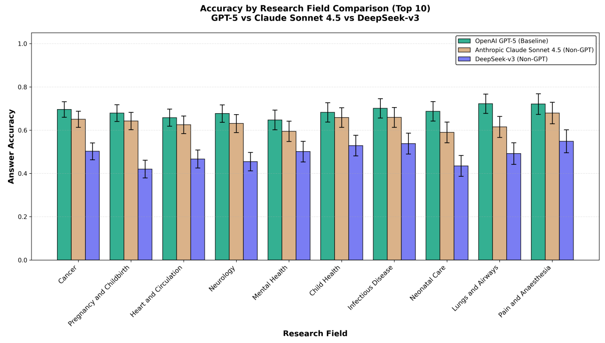

(c) Top 10 medical subfields by abstract count

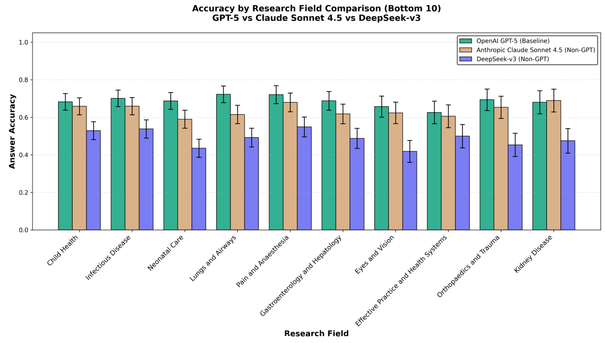

(d) Bottom 10 medical subfields by abstract count

Figure S1: Cross-model robustness analyses across evidence characteristics and clinical domains.

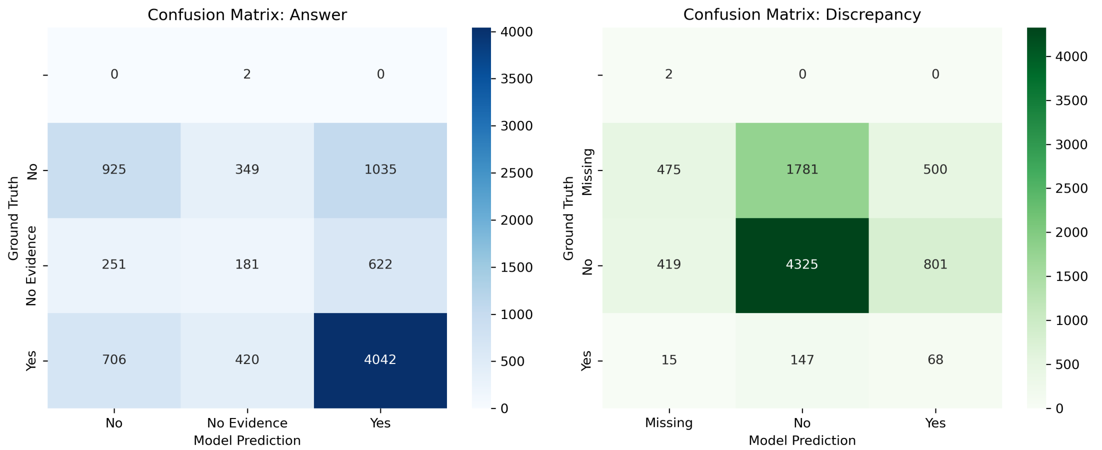

Figure S2: Confusion matrices for answer prediction and discrepancy detection using GPT-4o-mini.

the calibration set, and the second term estimates the apparent accuracy on the full evaluation set using generated answers.
